# First Web Space Reconstruction in Acquired Defects: A Literature-Based Review and Surgical Experience

**DOI:** 10.3390/jcm14103428

**Published:** 2025-05-14

**Authors:** Cesare Tiengo, Francesca Mazzarella, Luca Folini, Stefano L’Erario, Pasquale Zona, Daniele Brunelli, Franco Bassetto

**Affiliations:** Plastic Surgery Unit, Hand Surgery and Microsurgery Unit, Department of Neuroscience, University of Padova, 35121 Padova, Italy; francesca.mazzarella@aopd.veneto.it (F.M.); luca.folini@aopd.veneto.it (L.F.); lerariostefano@gmail.com (S.L.); pasquale.zona@aopd.veneto.it (P.Z.); daniele.brunelli@aopd.veneto.it (D.B.); franco.bassetto@unipd.it (F.B.)

**Keywords:** first web space, hand reconstruction, soft tissue reconstruction, first commissure

## Abstract

The first web space of the hand plays a fundamental role in daily hand function, facilitating crucial movements, such as pinching, grasping, and opposition. The structural anomalies of acquired defects of this anatomical region, whether secondary to trauma, burns, or post-oncological surgical resections, necessitate meticulous reconstructive strategies to ensure both functional restoration and aesthetic integrity. Given the complexity and variability of first web defects, a broad spectrum of reconstructive techniques has been developed, ranging from skin grafting and local flap reconstructions to advanced microsurgical approaches. This review comprehensively examines the existing literature on first web reconstruction techniques, analyzing their indications, advantages, and limitations. Additionally, it explores innovative techniques and emerging trends in the field, such as tissue engineering, regenerative medicine, and composite tissue allotransplantation, which may revolutionize future reconstructive strategies. The primary objective is to provide clinicians with an evidence-based guide to selecting the most appropriate reconstructive strategy tailored to individual patient needs. Furthermore, we incorporate our institutional experience in managing first web defects, highlighting key surgical principles, patient outcomes, and challenges encountered. Through this analysis, we aim to refine the understanding of first web reconstruction and contribute to the ongoing evolution of hand surgery techniques.

## 1. Introduction

The first web space is a definite anatomical region that is critical to the overall functionality of the hand. It is triangular in shape, with the apex pointing at the junction of the first and second metacarpal bone heads, and the base is made up of dorsal and palmar skin that spans from the first to the second metacarpophalangeal (MCP) joints. The thenar fibromuscular system makes up the inner part of the first web, and its external angle with the thumb in maximal abduction is typically 100°. This unique anatomy facilitates essential hand movements, such as abduction, opposition, and precision grip, which are indispensable in performing everyday tasks. Consequently, deformities or contractures affecting the first web space significantly compromise hand function and overall quality of life, necessitating surgical intervention [1,2]. The upper limb and hand, in particular, are frequently involved in traumatic lesions, which have a profound impact on the patient’s quality of life at a high socioeconomic cost [3]. Congenital conditions, such as syndactyly, often allow for reconstruction using adjacent tissues, whereas acquired defects—resulting from trauma, burns, tumors, or infections—pose more complex reconstructive challenges [4].

The literature reports a prevalence of hand scar contractures, ranging from 5% to 40%, in burned patients [5]. Scar contractures of the first web, in particular, are caused by a combination of skin deficiency, fascia and muscle fibrosis, and the underlying bony structure. It typically appears with the adduction and supination of the thumb, which alone accounts for 40–50% of total hand functionality [2,6]. The degree of contracture varies and Sandzén’s classification system (mild, moderate, and severe) provides a useful framework for determining the severity and corresponding surgical approach [7].

Reconstructive techniques must address both soft tissue deficits and underlying structural deformities. While traditional surgical methods, such as Z-plasty and local flaps, remain essential in mild-to-moderate cases, more extensive defects require advanced reconstructive solutions, including pedicled and microsurgical free flaps. Recent advances in biomaterials, vascularized composite allotransplantation (VCA), and three-dimensional (3D) bioprinting offer potential new frontiers for restoring complex hand defects.

This review examines the current state of first web space reconstruction, focusing on various reconstructive options, their indications, and long-term outcomes. We critically analyze the literature while integrating our institutional experience to offer insights into the practical applications of these techniques. By exploring the evolving surgical strategies for first web reconstruction, we aim to provide an academic foundation for improved clinical decision making.

## 2. Materials and Methods

This systematic review adhered to the recommendations of the Preferred Reporting Items of Systematic Reviews and Meta-analysis (PRISMA) 2009 guidelines. A computerized MEDLINE search was performed using the PubMed service of the U.S.A. National Library of Medicine accessed on 1 October 2024 (https://pubmed.ncbi.nlm.nih.gov). Three authors independently screened the articles and then selected and extracted data. This review is based on an extensive computerized analysis of the existing literature that was supplemented by our institutional experience in first web space reconstruction between 2010 and 2024. We systematically reviewed publications on reconstructive techniques, including skin grafts, local flaps, pedicled flaps, and microsurgical approaches. Key studies were selected based on their relevance, clinical outcomes, and contribution to current surgical practices. Additionally, we assessed innovative reconstructive techniques, including stem cell therapy and bioengineered tissue grafts. In addition, we retrospectively analyzed cases treated at our institution, including pediatric and adult patients requiring first web reconstruction. The selection criteria included patients with post-traumatic, post-burn, and post-oncologic resection web anomalies requiring surgical intervention. Surgical techniques were categorized based on defect complexity and reconstructive approach, with outcome measures including the first web space angle pre-and postoperatively, functional recovery, donor site morbidity, and complications. Data were analyzed using descriptive statistics and comparative studies where applicable.

### 2.1. Research Question

The inclusion criteria were as follows:

The a priori set inclusion criteria were randomized controlled trials, controlled clinical trials, controlled trials or systematic review, English full-text availability, and the most common reconstruction types for first commissure defects.

We admitted works including post traumatic cases, post-burns, and oncological defects. The titles and abstracts of the (k  =  233) articles found were independently screened by three researchers. A total of 44 articles were selected for full-text revision, which was conducted independently by two researchers.

The exclusion criteria were as follows: All articles presenting anatomical regions other than those including the first web space of the hand, congenital anomalies, and studies that incorporated a heterogeneous population (non-homogeneous etiologies of defects) were included only if each outcome could be extrapolated from individual patients.

### 2.2. Search Strategy

An electronic search was conducted from January 1985 up to June 2024 on the database PubMed (Medline). Although some older articles were included to capture historical reconstructive techniques, the majority of the selected studies were published between 2010 and 2020, reflecting current clinical practices and advancements in first commissure reconstruction.

Search strings were generated combining first commissure-related and treatment-related keywords using the Boolean operators “AND”. The types of treatment-related keywords themselves were combined with the function “OR”. The keywords used were proven for MeSH terms.

## 3. Results

At our institution, first web reconstruction procedures were performed in pediatric and adult patients using various techniques from the reconstructive ladder. The majority of pediatric cases were successfully managed with Z-plasty, local flaps, and skin grafting, whereas more extensive defects necessitated pedicled or microsurgical flaps.

Skin grafts and dermal substitutes: These techniques are employed primarily for superficial defects, and they yield satisfactory results when the underlying fascial structures are preserved. Split-thickness skin grafts from the foot instep are preferred due to their textural compatibility.

Local flaps and Z-plasties: These are first-line treatments for scar contracture release, particularly in burn sequelae. Multiple Z-plasty techniques, including 4-flap and 5-flap modifications, are employed to maximize web depth and length.

Pedicled flaps: Posterior interosseous artery flaps are used in cases requiring extensive soft tissue coverage, providing optimal pliability and skin texture match. These flaps are particularly advantageous due to their minimal donor site morbidity.

Microsurgical flaps: In complex reconstructions involving multi-tissue loss, free flaps, such as the anterolateral thigh (ALT) flap, are utilized. Superficial circumflex iliac artery (SCIP) flap are considered in smaller defects where a thinner and more pliable tissue is required.

The postoperative outcomes demonstrated a mean improvement in the first web space angle from 20° preoperatively to 75° postoperatively, with restored hand function in the majority of cases. Complications were minimal, with flap survival rates exceeding 90%. Additionally, emerging techniques, such as bioengineered scaffolds and tissue regeneration therapies, showed promise in preliminary clinical applications.

### 3.1. Local Flaps and Skin Grafts

#### 3.1.1. Skin Grafts and Dermal Substitutes (Table 1)

Skin grafting is a simple technique frequently used in first web contractures treatment. Split-thickness skin grafts are a good choice for restoring dorsal superficial defects, such as post-burn contractures. Skin can be picked up manually with a blade or with a dermatome depending on the extension of the defect and the donor site. The volar side of the wrist, the inner part of the arm, and the inguinal fold are the most frequent donor sites for small defects. The instep of the foot is also good donor site for palm reconstruction because it bears a small–medium area of glabrous skin with comparable thickness and texture to the recipient site [8]. The use of the dermatome is limited to a big-sized defect, where skin is usually obtained from the anterior part of the thigh. One of the main advantages of this technique is the possibility to obtain, when needed, a large patch of skin, adjustable in size and shape secondary to the defect, with limited morbidity of the donor site. Nonetheless, there are some drawbacks to skin-grafting procedures, such as a lack of sensation, inadequate thickness, and the risk of recurrence due to secondary contractures. In a review by Yuste et al., the authors reported that skin grafts, both split and full thickness, have a high rate of reintervention [3]. In some cases, especially when scarring affects the deep layer of the skin, the use of dermal substitutes in union with split-thickness skin graft is a valid option after contracture release. Dermal substitutes (which can be derived from ovine, swine, and bovine substrates), thanks to their high content of collagen, elastin, and hyaluronic acid, offer a scaffold that is colonized in two–three weeks by the fibroblasts and angiocytes of the patient. These matrices give good elasticity to the skin. After a period of two or three weeks, it is possible to cover the dermal substitute with a skin graft or heal the wound for a changed second intention moisturized dressing twice a week. Abbound and colleagues discussed their outcomes with dermal substitutes in patients with recessive dystrophic epidermolysis bullosa. While long-term functional outcomes for long fingers were favorable—with 57% of cases maintaining good function beyond three years—the results for first web space contractures were poor. Nearly all patients exhibited early recurrences of contractures within one year when treated with skin grafts. The authors reported a lower risk of recurrence when full-thickness skin grafts were used [9].

**Table 1:** This is a table describing the indications and techniques of skin grafts in soft tissue defects of the hand.

Author	Type of Study	Number of Cases/Studies	Indications	Technique
Ward, 1985 [8]	Case series	13	Hand burn contracture.	Split thickness graft from the plantar and volar instep donor sites.
Yuste, 2017 [3]	Review	29	First web contractures after full-thickness burns.	Skin grafts and dermal matrices, random flaps, pedicled fasciocutaneous flaps, free flaps, and other techniques.
Abboud, 2022 [9]	Retrospective review	18	Obliteration of the inter- digital spaces and adduction contracture of the thumb.	Fixation with Kirschner wires and covering with dermal substitutes or skin graft.

This high failure rate advises against the use of skin grafting in the first commissure and reinforces the preference for local or distant flap reconstruction, which offers more reliable, durable coverage and greater resistance to contracture recurrence.

#### 3.1.2. Local Flaps and Z-Plasties

For first web space small-sized contractures, common reconstructive options include local flaps and Z-plasties. Frequently, this kind of contracture affects children secondary to burns with hot surfaces, limiting the mobility of the fingers and the use of the hand. The availability and integrity of the surrounding tissues are crucial since these flaps depend on the skin that is immediately adjacent to the defect. There are many designs that could be used, according to the literature, but the most common flaps are the 4-flap Z-plasty, 2-flap Z-plasty, 5-flap Z-plasty, one Y-V advancement, and rotational flaps from the dorsum of the hand and index finger [2]. The 4-flap is made up of two 45° angle flaps that are transposed to gain length from a 90° angle flap. In order to increase both length and depth, it is also possible to contrapose two Z-plasties with a Y-V advancement in between [10].

#### 3.1.3. Local Axial and Perforator Flaps (Table 2)

Larger defects, extending on the dorsal or ventral skin, may require local axial or perforator flaps to bring healthy tissue in the first web space after contracture removal. Loco-regional flaps can have an axial or perforator vascularization pattern based on the dorsal metacarpal arteries. Dorsal metacarpal artery flaps and the distal-based dorsal metacarpal artery perforator flap (Quaba flap) are preferred due to their reliable blood supply [11]. In addition, the bilobed and V-Y advancement first dorsal metacarpal artery flaps are safe, simple, and versatile options that take advantage of the elasticity and mobility of the dorsal hand skin [12]. The first dorsal metacarpal artery (FDMA) flap was first described for thumb reconstruction [13]. It can be designed as a skin island to cover dorsal hand defects or transferred to the first web space. This allows for increased web width and a simple reconstruction with a consistent blood supply and minimal donor site morbidity [14]. Finally, Trimaille et al. published a case report in which they successfully remobilized a flap from a previous trauma repair site. The authors then transposed it to the contracted web space as an axial flap based on FDMA [15].

**Table 2:** This is a table describing the indications and techniques of local axial perforator flaps in soft tissue defects of the hand.

Author	Type of Study	Number of Cases/Studies	Indications	Techniques
Quaba, 1990. [11]	Anatomical study and case series	21	Resurfacing of web spaces and dorsal metacarpal and phalangeal skin defects.	The distally based dorsal hand flap.
Perera, 2014 [16]	Case report	1	First web space defect of the hand with a 3 × 2 cm skin deficit exposing neurovascular structures to the index finger.	Distally based dorsal metacarpal artery perforator flap (Quaba).
Doğan, 2014 [12]	Retrospective study	6	Burn wound adduction contractures of the first web space and acute wounds resulting from electrical burns and defects of the first web space and on the dorsum of the thumb.	V-Y advancement first dorsal metacarpal artery flap. Bilobed FDMA flap.
El Andaloussi, 2007 [14]	Case series	12	Skin defects on the dorsum of the distal phalanx of the thumbs on the dorsum of both the distal and proximal phalanges and on the volar aspect of the thumb.	The Foucher’s “kite-flap”.
Trimaille, 2015 [15]	Case report	1	Skin loss in a post-traumatic thumb defect in a 5-year-old child + secondary first web space narrowing.	First dorsal metacarpal artery flap associated with a toe-to-hand transfer + remobilization of FDMAF to open the first web space.

### 3.2. Locoregional Flaps (Table 3)

In extensive defects, the surgeon must consider the use of distant pedicled flaps or free flaps. Important contracture release of the first web, composite multi-tissue defects, exposed hardware, and poor-quality local skin are all conditions that necessitate distant or free flaps to restore the integrity of first commissure. In some cases, composite reconstruction, including tendon or nerve grafts, may be needed, especially after high-energy injuries. These are the most used pedicled flaps for first web reconstruction described in the literature.

**Table 3:** This is a table describing the indications and techniques of locoregional flaps in soft tissue defects of the hand plus arterial supply of locoregional flaps for first web reconstruction.

Author	Type of Study	Number of Cases/Studies	Indications	Techniques	Arterial Supply
Usami, 2017 [17]	Case series	13	Fingertip reconstructions.	Posterior interosseous artery perforator flap used for small defects.	Posterior interosseous artery.
Costa H, 2007 [18]	Anatomy study and retrospective review	102 clinical cases + 100 anatomical dissections	Large hand defects after crush–degloving injuries, burn contractures, or skin necrosis subsequent to chemotherapy, burns, or trauma. Soft tissue reconstruction of the first web space and the dorsal and palmar aspects of the hand, including the metacarpal–phalangeal joints and the dorsum of the thumb, as well as metacarpal reconstruction.	Posterior interosseous flap as either a fasciocutaneous island flap or an osteocutaneous flap.	Posterior interosseous artery.
Costa A, 2022 [19]	Systematic review	55	Post trauma, burn, and infection defects of the hand from the wrist to the fingers.	Reverse posterior interosseous flap.	Posterior interosseous artery.
Pagnotta, 2012 [20]	Anatomical study and clinical application report	2 clinical cases and 5 freshly injected cadavers.	Ulnar nonunion.	Dorsal distal radius vascularized bone graft pedicled on the posterior interosseous artery.	Posterior interosseous artery.
Zhang, 2013 [21]	Retrospective review	11	Soft tissue loss of the first web space and dorsum of the hand or palmum, thumb, and palm, the ulnar or dorsal palm, and the wrists and radial aspect of the thenar.	Reverse bipaddle posterior interosseous artery perforator flap.	Posterior interosseous artery.
Kai, 2013 [22]	Case series	12	Severe first web contractures after burn injury (chemical, thermal, and electrical).	Reverse posterior interosseous flap.	Posterior interosseous artery.
Vergara-Amador, 2015 [23]	Retrospective study	12	Defects on the volar or dorsal hand, the first web space, and the base of the long fingers.	Retrograde ulnar dorsal flap.	Dorsal branch of the ulnar artery.
Karacalar, 1999 [24]	Anatomic description and case report	2	First web and thumb IP joint contracture and multiple fractures and loss of the extensor tendons and dorsal skin.	Distally pedicled dorsoulnar flap.	Dorsal branch of the ulnar artery.
Uygur, 2009 [25]	Case series	36	Flexion contractures of palms and fingers after burn injuries, traumatic soft tissue loss, and tumor excision. Defects of the palm site and on the dorsum of the hand.	Dorsoulnar flap as either pedicle or free flap.	Dorsal branch of the ulnar artery.
Moody, 2015 [2]	Review	10	First web space contractures of different degrees.	Z-plasties, reverse posterior interosseous artery flap, free lateral arm flap, reverse radial forearm flap, and groin flap.	Radial artery, posterior radial collateral artery, etc.
Maan, 2017 [26]	Review	x	Coverage of moderate-size defects of the dorsal and volar hand.	Reverse forearm radial flap.	Radial artery.
Kaufman, 2005 [27]	Review	x	Medium- and moderate-sized defects of the dorsal hand.	Reverse forearm radial flap.	Radial artery.

#### 3.2.1. Reverse Radial Forearm (rRFF) Flap

The reverse radial forearm (RRFF) flap [26] is a well-established locoregional option for reconstructing soft tissue defects of the first commissure of the hand. It is based on the retrograde flow through the distal radial artery, relying on collateral circulation via the palmar arches and the ulnar artery. The flap is typically harvested from the volar aspect of the forearm, with ligation of the proximal radial artery, and it is rotated distally to cover defects involving the thumb base, first web space, or dorsal hand. It is particularly indicated in post-traumatic or post-oncologic reconstructions, burn contracture releases, or when free tissue transfer is contraindicated. Its main advantages include technical simplicity, the absence of microvascular anastomosis, and the thin, pliable tissue it provides—ideal for the contour-sensitive anatomy of the first commissure. However, disadvantages include the sacrifice of the radial artery, which may compromise hand perfusion in selected patients, and donor site morbidity, which often requires skin grafting [27]. Additionally, the flap may appear bulky or less aesthetically refined compared to free flaps, and it has a more limited arc of rotation.

#### 3.2.2. Reverse Posterior Interosseous Artery (rPIA) Flap

The rPIA flap is one of the best options for restoring defects from the wrist to the fingertips, and it has been widely employed in first web reconstructions [17]. The flap’s vascular supply is based distally on retrograde flow from the anterior interosseous artery (AIA) dorsal recurrent branch, which commonly anastomoses with the PIA 1.5–2 cm proximal to the distal radioulnar joint. The best methods to assess the presence of this vascular communication are preoperative manual Doppler and direct visualization. Nonetheless, the anastomosis can be absent or unstable in some circumstances, and the surgeon can convert the rPIA flap into a free flap [18]. The PIA branches from the common interosseous artery are approximately 4 cm distal to the lateral epicondyle. Its surface landmark is a line connecting the lateral epicondyle to the distal radioulnar joint with the elbow pronated and flexed at 90°. The cutaneous perforators are located in the septum between the extensor carpi ulnaris and extensor digiti minimi. The skin paddle is usually centered between the proximal and middle thirds of the forearm. Furthermore, some findings have indicated the most reliable perforators are more distal around the central region of the forearm [19]. The ligature of the anterior interosseous artery (AIA), which is proximal to the communicating branch with the posterior interosseous artery, has been demonstrated to increase the flap rotation arch, preserving both vascular sources and permitting to distally extend the reach of the flap [28]. The donor site can be closed directly up to 6 cm, or the skin can be grafted with great results and minimal donor site morbidity. The rPIA flap has acquired widespread popularity due to its several advantages: (1) it has a substantial vascular reliability; (2) it has thin and pliable skin with a good color and texture match; (3) it has a wide arch of rotation; (4) it does not sacrifice any major artery in the distal upper limb; and (5) it preserves lymphatic and venous drainages of the hand. The main disadvantage of the flap is venous congestion, which can be treated by loosening skin sutures or using leech therapy. Furthermore, supercharging the flap with additional venous anastomoses is an efficient method for reducing congestion [20]. Zhang et al. further addressed the necessity of reconstructing numerous subunits while limiting donor site morbidity. They divided the flap into two independent skin paddles, each with its own perforator. This chain-link flap enables for the simultaneous restoration of two separate hand subunits, whereas the kiss flap can resurface a single large defect while attaining primary donor site closure [21]. Finally, Kai et al. demonstrated that, with outstanding results, the rPIA flap could enhance the first web length by up to 260% when starting from a mean thumb to index angle of 78° [22].

#### 3.2.3. The Retrograde Dorsal Ulnar Pedicled Flap

The retrograde ulnar dorsal pedicled flap is a variant of the Becker’s ulnar flap and provides a safe and reproducible reconstructive method for hand and first web dorsal defects. The ulnar dorsal artery (UDA) proximal branch anastomoses with 2–3 perforating branches of the ulnar artery. This establishes a subcutaneous vascular plexus and enables for the design of a big flap with a distal pivot point. The distal branch of the UDA ensures reverse flow after the UDA is ligated at its origin [23]. In addition, authors have described other distant anastomoses that can give a strong vascular supply [24,25]. The main benefits of this flap are the preservation of the ulnar artery and low donor site morbidity. The reverse radial flap is an alternative to the UDA flap; however, it does compromise one of the two vascular axes of the hand and leaves a conspicuous scar on the patient’s forearm [2].

### 3.3. Free Flaps (Table 4)

There is literature that recommends using free flaps for first web reconstructions following severe contracture release or in the presence of exposed muscles, arteries, nerves, bones, and tendons. Furthermore, free flaps are required when the tissue loss extends beyond the first web space and involves the rest of the hand or forearm. In such instances, loco-regional flaps are no longer possible [3]. Currently, technology advancements and surgical procedure refinements have rendered the principle of the reconstructive ladder obsolete in favor of the reconstructive “tool box”. As a consequence, for many reconstructive surgeons, free flaps are now the first line of treatment, even for modest defects. Free flaps can overcome several issues of reconstruction since they can fit any size defect and bring as many good-quality tissues as are required to achieve the reconstructive goals [29].

#### 3.3.1. The Anterolateral Thigh (ALT) Flap

The ALT flap, defined as the ideal soft tissue flap by Wei et al. [30], is the predominant and most used flap for skin coverage by reconstructive surgeons. It can be harvested as a composite flap with a portion of vastus lateralis muscle, fascia lata, and, in rare situations, the iliac crest bone. These auxiliary tissues can be used to minimize dead space, recreate a gliding surface for tendons, and eventually repair the metacarpal bones [31]. The ALT flap is simple in harvesting technique and has a consistent anatomy; it has a long and wide diameter pedicle, and the anastomoses can be positioned distant from the zone of injury. Furthermore, donor site morbidity is minimal because the flap harvest involves no major or relevant anatomic structure [32,33]. The main concern with the ALT flap in hand reconstruction is its thickness, which can limit fine thumb movements when employed for first web reconstruction. Although further debulking treatments are safe and well tolerated by patients, the flap can be harvested as a thin or ultra-thin flap in the first place, or as a sandwich fascial anterolateral thigh (SALT) flap, as described by Cherubino et al. [34].

#### 3.3.2. Superficial Circumflex Iliac Artery Perforator (SCIP) Flap

The SCIP flap is another excellent option for hand skin coverage, and many surgeons across the world are using it as their first reconstructive technique. The SCIP flap provides a thin, pliable skin that is perfect for hand reconstruction. Furthermore, it has one of the greatest donor site outcomes because ordinary clothing and undergarments can readily conceal the scar. The flap’s typical drawbacks are excessive bulk in overweight patients and the need for subsequent debulking surgeries. These can be avoided by elevating the flap in the suprafascial plane or by raising it as a pure skin perforator flap [35]. The main disadvantages of the SCIP are that it is more difficult to dissect and requires more experience than the ALT flap.

#### 3.3.3. Foot Web Free Flap

The aforementioned flaps, despite being reliable and reproducible reconstructive solutions, do not guarantee a like-for-like repair of the initial web space. According to del Piñal et al., free foot first web flaps may be the best option for a like-for-like repair in post-traumatic hand and first web deformities [4]. A template of the first web defect can be translated to the foot web spaces using this technique. The size and nature of the hand deformity determine which foot web space will be used as the donor site. The first dorsal metatarsal artery is responsible for vascular supply, and, due to its length, it can be anastomosed with the deep branch of the radial artery or with the radial artery itself. The donor site is then either closed by creating a neosyndacty or repaired with dermal substitutes or skin grafts. The flap completely matches the 3D structure of the initial web with a good texture and color match for small–medium defects. In contrast to other free flaps, there is no bulging and the skin folds inward during adduction, mirroring the biomechanical features of the native first web. As the donor artery diameter spans from 0.5 to 1 mm, advanced microsurgical skills are required. Furthermore, donor site morbidity may be bothersome for some patients. The formation of a neosyndactily at the donor site is normally well tolerated for the second web flap, but a painful hyperkeratosis may occur in the event of a skin graft for donor site closure.

**Table 4:** This is a table describing the indications and techniques of free flaps in soft tissue defects of the hand.

Author	Type of Study	Number of Cases/Studies	Indications	Techniques
Yuste, 2017 [3]	Review	29	First web contractures after full-thickness burns.	Skin grafts and dermal matrices, random flaps, pedicled fasciocutaneous flaps, free flaps, and other techniques.
Meky, 2013 [31]	Case series	3	Complex defects of hand involving different tissues (bone, tendon, and skin).	Composite anterolateral thigh flap.
Miller, 2016 [29]	Review	x	Various hand defects.	Reverse homodigital island flap, reverse cross-finger flap, radial artery perforator flap, groin flap, lateral arm free flap, posterior interosseous artery perforator flap, anterolateral thigh flap, and first dorsal metacarpal artery flap.
Wei, 2002 [30]	Retrospective study	672	Multitissutal reconstruction of the whole body: head/neck, upper limb, lower limb, and trunk.	Anterolateral thigh flap.
Cai, 2021 [32]	Prospective series	14	Soft tissue defects and major forearm vascular axis of the hand.	Anterolateral thigh flap.
Wang L [33]	Retrospective	6	Hand soft tissue defects.	Deep anterolateral thigh fascial flaps associated to skin grafts.
Cherubino, 2017 [34]	Retrospective	11	Head/neck reconstruction. Indications can be extended to any other body area.	Grafted thin adipofascial anterolateral thigh flap.
Narushima M. [35]	Case series	6	Soft tissue defects, including middle finger, little finger, thumb, dorsum, and palmar hand.	Superficial circumflex iliac artery pure skin perforator-based superthin flap.
Del Piñal, 2015 [4]	Case series	9	Post-traumatic and post-infective hand web contractures.	Foot web free flaps for single-stage reconstruction of hand webs.
Jeon, 2017 [36]	Case series	10	Small-sized hand defects of the fingers and first web space.	Anconeus free flap.
He, 2017 [37]	Case series	15	Moderate-sized hand defects: palm, dorsum, finger, and first web reconstructions.	Superficial lateral sural artery perforator flap.
Wolff, 2011 [38]	Anatomical study	42	Moderate-sized intraoral defects.	Superficial lateral sural artery free flap.
Lin, 2011 [39]	Case series	14	Small-to-medium-sized hand defects involving fingers, dorsal hands, palms, and wrist.	Medial sural artery perforator flap.
Xie, 2007 [40]	Case series	7	Soft tissue defects of the hand, mainly the dorsum of the hand.	Medial sural artery perforator flap.

### 3.4. Minor Free Flaps

#### 3.4.1. Anconeus Flap

Jeon et al. advocated the use of the anconeus muscle free flap in a group of ten patients [36]. This flap has the benefit of being harvested within the same surgical field of the affected hand and leaving with small or no functional deficits following its harvest. The recurrent posterior interosseous artery provides vascular inflow. The mean pedicle length was 1.6 cm (range: 1.0 to 2.1 cm), and the artery diameter was about 1 mm. Despite the good results achieved by the authors, interposition vein grafts are frequently necessary to reach the recipient vessel. Finally, the lack of a skin island necessitates the use of a skin graft for wound closure at the recipient site.

#### 3.4.2. Superficial Lateral and Medial Sural Artery Flap

In 2017, He et al. published a case series of 15 patients treated with the superficial lateral sural artery perforator (SLSAP) flap for the repair of moderate-sized hand wounds, including the first web space [37]. Notably, in a 2012 anatomical research, the superficial lateral sural artery was present in 36 of 42 specimens (85.8%). However, only 25 of 36 arteries (69.4%) were suitable for free flap harvest [38]. When the lateral vessels are absent or unreliable for microsurgical anastomosis, the surgeon must convert the flap into the more popular superficial medial sural artery perforator (MSAP) flap, extending the incision medially and dissecting until the medial sural artery and its perforators are exposed. The flap is soft and thin, and suitable for medium-sized defects, when donor site morbidity is low, and when the harvest technique is relatively simple. The anatomy of the MSAP, on the other hand, is more constant, and surgeons tend prefer it to its lateral counterpart [39,40]. Finally, both flaps have a medium-length pedicle (8 cm on average for SLSA, 10 cm for MSAP [38,41]) and moderate-sized skin paddles (4 × 6 cm on average for SLSA, 10 × 5 cm for MSAP [38,41]), making them excellent for moderate-sized defects with anastomosis close to the zone of injury.

#### 3.4.3. Preferred Recipient Vessels for Microvascular Anastomosis (Table 5)

In microvascular reconstruction of the first commissure of the hand, the choice of recipient vessels is guided by several factors, including anatomical proximity to the defect, vessel caliber compatibility with the flap pedicle, ease of dissection, and the quality of blood flow. One of the most commonly used vessels is the radial artery, particularly its superficial branch located in the anatomical snuffbox or the deep segment in the distal forearm. This vessel is frequently selected due to its accessibility and reliable caliber, making it ideal for reconstructions involving the thumb or first web space. Dorsal branches of the radial artery, located on the dorsum of the hand near the anatomical snuffbox, are another useful option, especially when a dorsal approach is planned, as they allow preservation of the main radial artery trunk. The superficial palmar arch, situated in the palm, may be employed in palmar-based reconstructions, particularly when the arch is exposed or involved in the defect. Less commonly, the princeps pollicis artery, arising from the deep palmar arch and running along the ulnar aspect of the thumb, may be used in selected deep reconstructions, though its access is limited by its deeper location. For venous drainage, the subcutaneous dorsal venous network is typically preferred due to its easy accessibility, though palmar veins may be considered in specific cases. The distal cephalic vein, located on the radial side of the forearm or wrist, offers reliable size and flow, and it is often utilized during dorsal approaches. When selecting recipient vessels, surgeons must also consider the surgical approach, as dorsal or palmar access affects vessel exposure; the size match with the flap pedicle; any history of trauma or scarring in the operative field; and the orientation of the flap inset. All of these eventualities can impact the pedicle’s reach and routing.

**Table 5:** This is a table describing the preferred recipient vessels or anatomical locations for microvascular anastomoses in first web space reconstruction.

Recipient Vessel	Anatomical Location	Typical Indication/Use
Radial Artery (Superficial/Deep Branch)	Lateral wrist or anatomical snuffbox.	Primary choice for first web space due to accessibility and good caliber.
Dorsal Radial Artery Branches	Hand dorsum next to anatomical snuffbox.	Suitable for dorsal approaches and avoids sacrificing the main radial artery.
Superficial Palmar Arch	Palm, proximal to the base of the fingers.	Useful when palmar access is preferred and may be exposed during volar reconstructions.
Princeps Pollicis Artery	Along ulnar side of the thumb, from deep palmar arch.	Rarely used though suitable for deep, central reconstructions involving the thumb base.
Dorsal Venous Network	Subcutaneous veins on dorsum of the hand.	Common for venous drainage and easily accessible.
Palmar Venous Network	Subcutaneous veins on palm.	Less frequently used and may be suitable in volar approaches.
Cephalic Vein (Distal)	Radial forearm or wrist.	Reliable venous recipient, often used when dorsal access is undertaken or for longer pedicles.

## 4. Discussion and Future Directions

Our findings align with the existing literature on first web reconstruction, reaffirming the importance of individualized treatment strategies. The reconstructive approach must consider defect severity, tissue quality, and functional demands. While skin grafts remain a viable option only for superficial and small defects, their tendency for secondary contracture limits long-term effectiveness. Dermal substitutes offer improved outcomes but are still associated with recurrence risks, especially in dynamic areas like the first web space.

Local flaps and Z-plasty techniques provide effective solutions for mild-to-moderate contractures, offering the advantage of single-stage reconstruction with minimal donor site morbidity. However, deeper structural compromise necessitates the use of pedicled flaps, such as the posterior interosseous artery flap, which has demonstrated superior results in providing well-vascularized, pliable tissue with minimal complications. Microsurgical flaps remain indispensable for extensive defects involving composite tissue loss. The anterolateral thigh (ALT) flap has emerged as our preferred option due to its adaptability, large skin paddle, and consistent vascularity, though secondary debulking procedures are often required to optimize hand function.

Recent innovations in microsurgery, such as super-microsurgery and perforator flaps, have enhanced surgical precision and minimized donor site morbidity. The integration of bioengineered scaffolds, tissue engineering, and stem cell applications presents an exciting frontier in reconstructive surgery. These advancements hold promise for improving long-term outcomes, reducing secondary contractures and potentially eliminating the need for extensive donor sites. Furthermore, the development of nerve regeneration techniques and functional muscle transplantation could play a pivotal role in restoring intricate hand movements in severe first web defects [42,43,44].

Despite these advancements, challenges remain in achieving optimal functional and aesthetic results, particularly in complex cases involving extensive scarring, bone loss, or compromised vascularization. Future research should focus on refining flap designs, optimizing donor site morbidity, and improving functional rehabilitation post-reconstruction. The integration of 3D bioprinting technology, regenerative medicine, and vascularized composite allotransplantation (VCA) [42] may offer groundbreaking solutions for hand reconstruction in the coming decades.

### 4.1. Our Experience

At our institution, a Level II trauma center, we performed numerous first web space reconstructions between 2010 and 2024, addressing acquired defects resulting from trauma, oncologic resections, and burn contractures. Our approach has evolved over the years to incorporate evidence-based surgical techniques and patient-centered rehabilitation protocols.

#### 4.1.1. Patient Demographics and Case Distribution

Among our patients, the mean age was 44 years, with 80% presenting post-burn contractures and 20% suffering from traumatic crush injuries. Pediatric cases were primarily managed with Z-plasties, local flaps, and skin grafting, whereas adult patients with severe contractures or composite defects required more extensive interventions.

#### 4.1.2. Surgical Techniques and Outcomes

**Figure 1:**
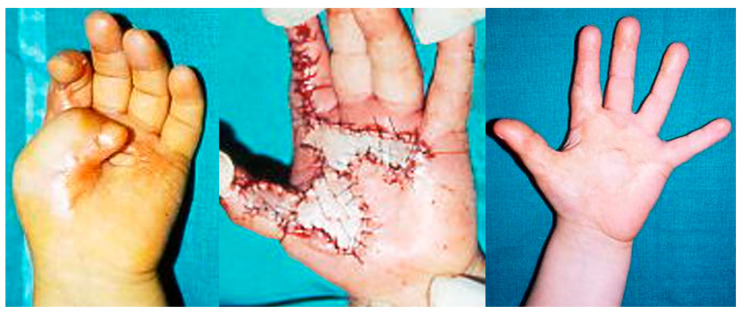
These images illustrate the removal of scar contractures of the first web space and repairs with a skin graft in a pediatric hand.

Skin grafts and dermal substitutes: In cases where contractures were limited to superficial layers, we successfully employed split-thickness skin grafts, which were often combined with dermal substitutes to reduce recurrence. The donor sites were the inguinal fold, volar side of the wrist, and the anterior side of the thigh for extended defects. Our findings support the literature, indicating that full-thickness skin grafts have superior long-term durability compared to split-thickness grafts alone. In some cases, when contracture affected the superficial fascia, we decided to use a dermal substitute, with skin grafted after three weeks, thereby obtaining a good final texture and pliability (Figure 1).

**Figure 2:**
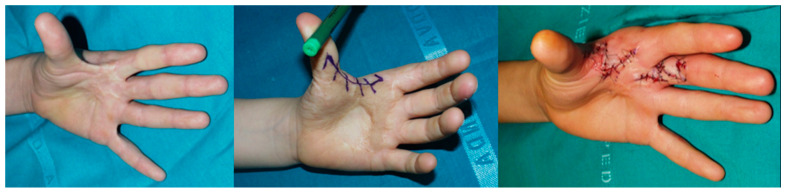
A Z-plasty illustrated as a workhorse technique for first web space release.

Z-plasty and local flaps: Z-plasties remain our first-line surgery for mild-to-moderate small-sized contractures, particularly for burn sequelae. By combining multiple Z-plasties with Y-V advancement techniques, we achieved significant first web space widening while preserving mobility. This approach resulted in a mean postoperative angle increase from 20° to 75°. Donor site was closed by primary intention (Figure 2).

Pedicled flaps: In ten cases involving moderate-to-severe contractures, we utilized posterior interosseous artery flaps and reverse forearm flaps. The posterior interosseous artery flap demonstrated excellent pliability and integration, with a high success rate and minimal donor site morbidity.

Microsurgical flaps: For large, complex defects, we employed free flaps in eight cases, with seven anterolateral thigh (ALT) flaps and one second toe transfer. Microsurgical reconstructions were particularly beneficial for cases involving severe scarring, tendon exposure, and bone loss. The ALT flap provided durable coverage with a high survival rate, though secondary debulking procedures were needed in 40% of cases.

Among our microsurgical cases, six were performed as emergency procedures for mangled hand injuries, requiring meticulous preoperative planning and intraoperative execution. Our experience supports the growing trend of using free flaps as a primary option for extensive defects rather than following the traditional reconstructive ladder approach (Table 6).

**Figure 3:**
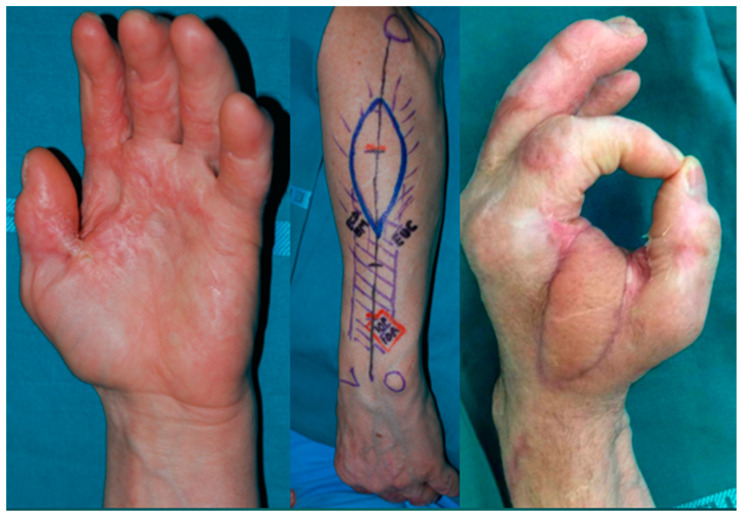
A case of a first web space post burn contracture managed with reconstruction through a posterior interosseous flap.

We performed a posterior interosseous flap in eight cases. We consider this flap a first option in first web space reconstruction thanks to its good pliability and match in skin texture (Figure 3).

In particular, its low thickness makes this flap easily adapt to the fold between the first and second digit without bulkiness issues. The flexibility of the dorsal skin of the forearm allows, once the flap is turned in its final position, the maintenance of the range of motion of the digit, preventing secondary contractures. The donor site can be closed by primary intention if the flap width is under 6 cm, limiting the morbidity of the forearm.

**Figure 4:**
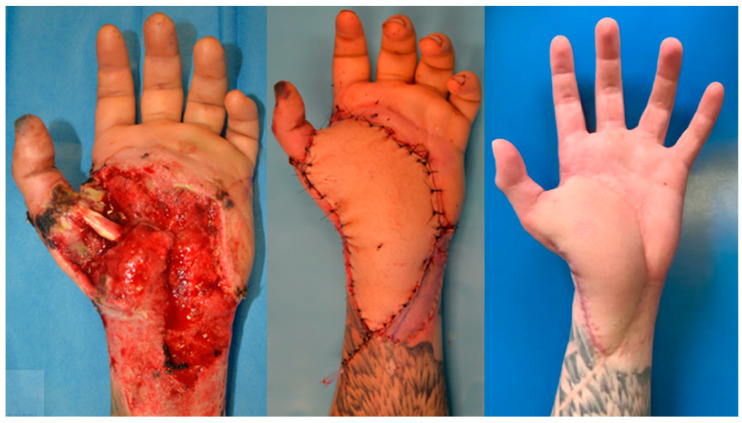
Use of ALT flaps to repair big defects of first commissures and the palmar side of the hand after an explosion injury.

In cases where the defect includes more than two units of the hand or is a composite (muscle and tendon loss or bone exposure) and cannot be covered with a locoregional flap, we usually use a microsurgical flap. We fully concur with Wei et al. that the ALT flap is the best choice for upper and lower limb reconstruction, as well as for head and neck reconstruction, at our hospital. The ALT flap is a robust flap with a long pedicle that can be anastomosed with the radial artery’s deep branch at the anatomic snuffbox or with the radial artery itself. It is compatible with skin texture and color, and the flap harvest can be performed by a different surgical team for timing optimization (Figure 4).

We prefer to harvest the fascio-cutaneous flap and plan a secondary debulking later on for the patient. In our experience, single- or dual-stage reconstructions produce comparable results. We experienced the use of this flap in urgency reconstruction for hand trauma and in elective surgery for post scarring contractures of the first web space.

In our experience, we consider the SCIP flap a second option for soft tissue restoration of the first web space and hand. It has a much shorter pedicle and smaller caliber than the ALT flap. This poses a challenge when dealing with complex defects with a distant site of anastomosis. Furthermore, there is limited tissue available in the groin for composite and chimeric flaps. When dealing with small–medium-sized lesions that do not affect the underlying structures, we recommend employing the SCIP flap.

**Figure 5:**
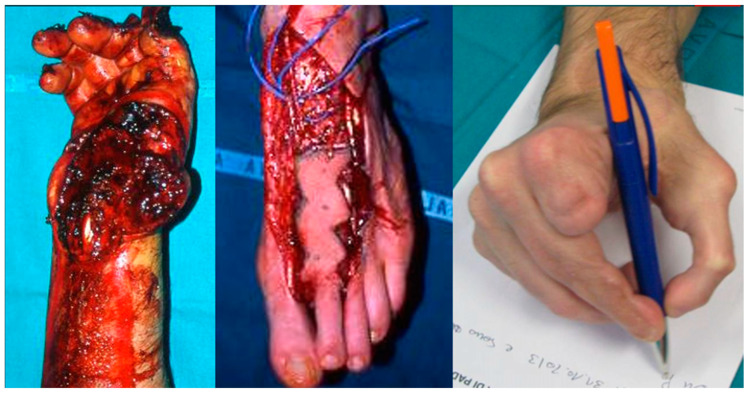
Pictures of a II toe transfer for reconstruction of the I digit and web commissure after workplace trauma.

When the first digit of the hand is fully damaged it is necessary to restore the use of the hand with the transfer of the hallux or the second toe (Figure 5). We used a second toe transfer in an emergency setting to recreate the first digit of the hand, obtaining a good functionality of the hand.

#### 4.1.3. Complications, Flap Survival, and Long-Term Functional Outcomes

In our clinical series, which includes both locoregional and free flaps for reconstruction of the first web space, we observed consistently high flap survival rates and satisfactory functional outcomes. Among the 30 locoregional flaps performed, the survival rate was 96.7%, with minor complications occurring in 10% of cases. These included superficial epidermolysis, marginal venous congestion, and minor wound dehiscence, all of which were resolved with conservative management. One flap (3.3%) showed partial distal necrosis and required secondary revision under local anesthesia. Aesthetic or functional reoperations, such as contouring or secondary contracture release, were performed in three patients (10%).

In our cohort of 12 free flaps, predominantly involving the anterolateral thigh (ALT) flap and lateral arm flap, complete survival was achieved in 11 cases (91.7%). One flap was lost due to arterial thrombosis on postoperative Day 2, necessitating removal and subsequent reconstruction with a pedicled flap. Minor complications, such as hematoma or donor site seroma, occurred in two patients (16.7%) and were managed successfully. Secondary debulking procedures were performed in three cases (25%) to optimize contour and functional mobility of the thumb–index web space. Importantly, the use of free flaps allowed for reliable coverage in complex cases involving extensive soft tissue loss, exposed tendons, or the need for composite reconstruction, with overall patient satisfaction remaining high.

Overall, our patients demonstrated significant functional improvements, with a majority regaining near-normal thumb–index opposition and grip strength. Long-term follow-ups indicated sustained flap viability, with minimal late-stage complications, such as secondary contracture or bulkiness in ALT reconstructions. Patients who underwent SCIP flap reconstructions reported high satisfaction due to the thin and pliable tissue characteristics, though the technique was less frequently utilized due to the shorter pedicle length.

### 4.2. Future Directions in Clinical Practice

Based on our institutional findings and a review of the literature, we advocate for an individualized, defect-specific approach in first web reconstruction. The combination of advanced microsurgical techniques, improved biomaterials, and optimized rehabilitation protocols has significantly enhanced patient outcomes. Future research at our institution will focus on incorporating regenerative medicine approaches, including stem cell therapy and scaffold-based tissue engineering, to further improve reconstructive options.

In conclusion, first web space reconstruction remains a highly specialized and evolving field in hand surgery. Our institutional experience corroborates the broader surgical literature, emphasizing the importance of technique selection, early intervention, and multidisciplinary management to achieve optimal results

## 5. Conclusions

The first web space is essential for hand function, and its reconstruction requires a tailored approach based on defect characteristics. The choice of technique should balance functional restoration, aesthetic outcomes, and long-term durability. Continued advancements in surgical techniques and bioengineering will further enhance reconstructive outcomes for first web anomalies.

## Figures and Tables

**Table 6:** This is a table describing the cases treated, from 2010 to 2023, in our hospital for first web contractures and the flap that was chosen for reconstruction.

Clinical Data	Findings
Number of patients	18 patients; mean age 44 years
Types of pedicled flaps	8 posterior interosseous flaps, 3 radial forearm flaps
Types of microsurgical flaps	9 anterolateral thigh flaps, 1 second toe transfer
Urgent flap procedures	6 flaps for mangled hand injuries
Mean preoperative first web space angle	20 degrees
Mean postoperative first web space angle	75 degrees

## Data Availability

The data presented in this study are available on request from the corresponding authors due to institutional policy.

## Abbreviations

The following abbreviations are used in this manuscript:

MCP	Metacarpophalangeal joints
VCA	Vascularized composite allotransplantation
SCIP	Superficial circumflex iliac artery perforator
ALT	Anterolateral thigh flap
rPIA	Reverse posterior interosseous artery flap
FDMA	First dorsal metacarpal artery
AIA	Anterior interosseous artery
UDA	Ulnar dorsal artery
SALT	Sandwich fascial anterolateral thigh flap
SLSAP	Superficial lateral sural artery perforator
MSAP	Sural artery perforator flap
DOAJ	Directory of open-access journals
TLA	Three-letter acronym
LD	Linear dichroism
